# Understanding and Addressing Problems in Research Collaboration: A Qualitative Interview Study From a Self-Governance Perspective

**DOI:** 10.3389/frma.2021.778176

**Published:** 2022-02-03

**Authors:** Florian Meißner, Carina Weinmann, Gerhard Vowe

**Affiliations:** ^1^Department of Culture, Media, Psychology, Macromedia University of Applied Sciences, Cologne, Germany; ^2^Department of Social Sciences, Heinrich Heine University of Düsseldorf, Düsseldorf, Germany; ^3^Center for Advanced Internet Studies (CAIS), Bochum, Germany

**Keywords:** research collaboration, collaborative research, collaboration problems, solutions, self-governance, club theory, commons theory, expert interviews

## Abstract

For collaborative research to be successful, understanding and solving collaboration problems is of paramount importance. However, theory-driven research on this issue at a general level is scarce. Drawing from two micro-oriented approaches (i.e., club theory and commons theory) and relying on self-governance as the basic principle for addressing collaboration problems, we aim to develop theoretically informed, concise and generalizable catalogs of problems and solutions based on the experiences and expectations of research collaboration participants. A series of expert interviews (*N* = 18) were conducted with leading researchers in Germany. Seven typical problems (e.g., lack of commitment or fairness) and 12 possible solutions (e.g., continuous evaluation or creating cognitive common ground) that can be applied within the self-regulatory framework were identified. The results provide a useful framework to further investigate problems and solutions as well as interlinkages between the two, and to improve research collaboration.

## Introduction

The growing importance of collaborative research in science can be observed, for example, in the increasing number of co-authored publications (Gross et al., 2017; Hackett et al., 2017). As research collaborations (RCs) are one of the most efficient ways to tackle complex research issues, they have become the most common form of scientific work (see e.g., Katz and Martin, 1997; Beaver, 2013; Bozeman and Youtie, 2017; Wagner, 2019). However, like any other type of collaboration, RCs face specific problems, including personal differences between participants or ineffective communication (e.g., Youtie and Bozeman, 2014; Bozeman et al., 2016). If such problems are not addressed, substantial risks to productivity and to the success of scientific work may result (e.g., Bozeman et al., 2016; Sacco, 2020). While there are studies which provide comprehensive lists of factors that are either detrimental or supportive in the context of RC for specific disciplines (e.g., Bozeman et al., 2016; Volk, 2021), this issue has not been addressed for RC in general. Furthermore, we do not see an integrative theoretical basis from which potential problems and appropriate solutions might be derived. In our view, such a basis is crucial: While pragmatic reactions to problems seem to be popular and might be useful on an everyday basis, they do not allow for the generalization of researchers' experiences with collaboration problems and of solutions which can be relied upon beyond single contexts. We therefore aim to address these research gaps by systematizing and generalizing the problems typically faced by researchers in RC, and by gleaning researchers' suggestions to address and solve these problems.

The present study is based on the assumption that problems arise from inside a RC and that these problems need to be addressed by those who are involved. As such, we focus not on external problems such as changes to national science policy, but rather on internal problems. In our understanding, a collaboration problem arises if a substantial proportion of participants perceive that the current collaboration practice deviates from their expectations of how collaboration should look. Our focus lies on normative expectations: In contrast to empirical expectations, which describe what individuals expect others to actually do, normative expectations refer to how individuals think others should behave (see Bicchieri, 2006). For instance, a researcher may perceive that his/her collaborators are not behaving as ethically as expected (e.g., Sacco, 2020). Accordingly, our first research question is as follows:

RQ1: Which problems do researchers perceive with regard to RC?

Scholars often refer to external forces such as science policy makers, state funding agencies, or bureaucratic mechanisms in order to conceptualize the governance of science (e.g., Shrum et al., 2007; Bora, 2012; Hackett et al., 2017; Gläser, 2019). However, we assume that collaboration problems must be solved within the very context of RC, which is why we focus on internal governing mechanisms. We thus suggest self-governance as the basic principle for solving collaboration problems. Moreover, we understand that solutions to collaboration problems encompass approaches that participants consider to be useful for converging the current practice of collaboration toward their expectations. In this sense, solutions can be seen as the means with which to address or even prevent collaboration problems. Therefore, our second research question is:

RQ2: Which problem solutions do researchers within RC perceive as useful?

The answers to these research questions should be of theoretical relevance by contributing to a more thorough understanding of RC: We aim to develop theoretically informed and empirically based catalogs of collaboration problems and possible solutions, which are based on the experiences and expectations of researchers and may be used as a basis for further empirical research. Concerning the practical relevance, the answers should enhance the productivity and efficiency of collaborative research: The greatest possible benefits (e.g., gain of scientific knowledge, technological innovations) with the lowest possible consumption of resources might be achieved. We begin with a review of the current state of research, which shows a rather fragmented picture of problems and solutions as well as a rather thin theoretical basis. Subsequently, we provide an integrative micro-theoretical basis upon which to understand collaboration problems and identify problem solutions. After explaining the method and sample of our qualitative interview study, we generate differentiated catalogs of collaboration problems and solutions to these problems. Lastly, we outline how our findings contribute to painting a clearer picture of the fragmented research on problems and problem solutions in RC, and how they can be used by leading researchers and research managers.

## State of Research: Problems and Solutions in the Context of Research Collaboration

We understand research collaborations (RCs) as inter- or intra-organizational teams of researchers who work together in order to achieve a common research objective, while their research project is limited in time and mostly funded by external resources (see e.g., Katz and Martin, 1997; Bozeman et al., 2013; Kosmützky, 2018). In contrast to larger collaborations, such as those examined in the prominent study by Shrum et al. (2007), the associations we address only exhibit a low level of bureaucracy. They consist of a smaller or greater number of participants, who form a community comprising all individuals who are scientifically involved in a collaboration, i.e., the leadership (speakers and coordinators), principal investigators, as well as further participants (postdocs and doctoral students). Beyond science and technology studies as the primary domain, RC has been studied by a variety of other disciplines, including the economic sciences (e.g., Ankrah and AL-Tabbaa, 2015; Baurmann and Brennan, 2016), sociology (e.g., Lewis et al., 2012; Costa, 2014), psychology (e.g., Cummings and Kiesler, 2007; Akkerman et al., 2012), or communication science (e.g., Wöhlert, 2020; Volk, 2021). A wide range of questions and actor constellations have been investigated, from university-industry collaborations (e.g., Mascarenhas et al., 2018; Vick and Robertson, 2018), through collaborations between universities or research centers (e.g., Adams et al., 2005; Muriithi et al., 2018), to RC that also includes further actors like public governance institutions (e.g., Kezar, 2005; Yang, 2018).

### Insights Into Collaboration Problems

Previous research has identified diverse problems that occur in collaborative research. Most of all, studies mention various forms of differences that can lead to collaboration problems, for example:

- disciplinary differences (e.g., Cummings and Kiesler, 2008),
- different institutional logics (e.g., Cummings and Kiesler, 2007; Bjerregaard, 2010; Garcia et al., 2019),
- different perspectives, styles of working, and priorities of participants (e.g., Adler et al., 2009; Bozeman et al., 2016; Garcia et al., 2019),
- different educational contexts (e.g., Goddard et al., 2006; Volk, 2021),
- gender and cultural differences (e.g., Bozeman and Gaughan, 2011; Abramo et al., 2013; Dusdal and Powell, 2021).

Additionally, ineffective or insufficient communication (e.g., Barnes et al., 2002; Cohen et al., 2011; Wöhlert, 2020) and status and role conflicts (e.g., Hackett, 2005; Bendersky and Hays, 2012; Youtie and Bozeman, 2014) are regarded as prominent problems. A lack of experience and commitment of participants (e.g., Barnes et al., 2002; Gaskill et al., 2003; Bozeman et al., 2016) and unprofessional or inefficient leadership and management (e.g., Adler et al., 2009; Bozeman et al., 2016; Volk, 2021) are further recurrent problems. Less frequently named examples are a lack of sustainability in funding (Goddard et al., 2006; Adler et al., 2009; Volk, 2021), the relation between individual costs and benefits (Berlemann and Haucap, 2015), geographical distance (Goddard et al., 2006; Cummings and Kiesler, 2008; Volk, 2021), the size of the research team (e.g., Cummings and Kiesler, 2008; Cummings et al., 2013), a high level of bureaucracy in institutions (e.g., Muriithi et al., 2018), or unforeseeable risks like the premature disclosure of results by participants (e.g., Hoecht, 2004; Hackett et al., 2017; Garcia et al., 2019).

### Insights Into Solutions to Collaboration Problems

Previous research also suggests a variety of individual solutions to collaboration problems. The most frequently mentioned solutions by far include competent leadership and management (e.g., Vonortas and Spivack, 2006; Schützenmeister, 2010; Volk, 2021) and effective and continuous communication, negotiation, and networking (e.g., Jeffrey, 2003; Vicens and Bourne, 2007; Luo and Omollo, 2013). Further prominent examples include fostering commitment, trust, and a good relationship between collaborators (e.g., Chompalov and Shrum, 1999; Shrum et al., 2001, 2007; Bruneel et al., 2010) and the provision of funding, incentives, and rewards as well as motivating participants (e.g., Heller and Michelassi, 2012; Currie-Alder et al., 2018; Wagner et al., 2019).

Furthermore, working with previous collaboration partners (Chapman et al., 2018; Liang and Liu, 2018; Hewitt-Dundas et al., 2019), optimal team size and composition (e.g., Porac et al., 2004; Holl and Rama, 2019; Mirnezami et al., 2020), and working with partners with similar styles of working and objectives (e.g., Hara et al., 2003) are also regarded as effective, albeit less frequently. Some studies also highlight the definition of clear objectives (e.g., Bjerregaard, 2009; Begun et al., 2010; Lee and Mitchell, 2011), project planning and monitoring (e.g., Segalla, 1998; Barnes et al., 2002; Morandi, 2013), as well as an efficient and appropriate division of labor (e.g., Raadgever et al., 2012; Jeong and Choi, 2015; Haeussler and Sauermann, 2020). Individual studies also point, for example, to the use of interdisciplinary working methods (Scanlon et al., 2019), to the recognition and bridging of individual differences such as race or gender (e.g., Ettorre, 2000; Bammer, 2008), or to a strong group identity (e.g., Jackson, 2011).

This review demonstrates the variety of individual approaches to collaboration problems and their solutions. However, the overall picture is rather fragmented: Research has often focused on individual problems and solutions instead of targeting the “whole picture.” And those exceptions which provide comprehensive lists of challenges for collaborative research and ways to solve them focused on specific disciplines like the STEM fields (Bozeman et al., 2016) or communication science (Volk, 2021). Moreover, while scholarship on RC is constantly growing, just as the phenomenon itself (e.g., Hackett et al., 2017; Mascarenhas et al., 2018), the theoretical basis of this line of research is rather thin and extremely diverse. This is problematic insofar as we are left without a thorough theoretical understanding of why and how which kinds of collaboration problems emerge. Furthermore, without a solid theoretical basis, we can hardly determine which solutions might be most effective and who should be held responsible. To summarize, as yet, systematic and generalizable catalogs of problems and solutions that are interlinked and coherently derived from an integrative theoretical basis and applicable to RC in general are lacking. As such catalogs should facilitate the diagnosis of collaboration problems and how they are dealt with, we aim to address this issue and will elucidate our theoretical perspective in the following section.

## Theoretical Background: Club Theory and Commons Theory

Our study is based on the assumption that collaborative research, i.e., the processes, rules, and procedures, is mainly driven by the involved researchers themselves. Accordingly, central problems in collaborative research arise from individual perceptions, interactions, and relationships within a research team itself, and therefore have to be solved within this very group of researchers. Thus, in contrast to studies that take a meso perspective (e.g., Shrum et al., 2007), our emphasis on the individuals involved in collaboration calls for a micro-theoretical view. The core of the theoretical framework is thus an approach which combines two decidedly micro-oriented perspectives: club theory (Buchanan, 1965) and commons theory (Ostrom, 1990, 2005). These theories allow us to understand collaboration problems and potential solutions based on the individual perspectives and the tension that exists between individual and common interests. Both theories have proven to be valuable in other contexts. For example, club theory has been used to explain the functioning of voluntary programs (Prakash and Potoski, 2009), while commons theory has been applied to traditional topics like fishery and forestry and to newer ones such as climate change and digital commons issues (van Laerhoven and Ostrom, 2007). Both theories promise conceptual clarity in our context: On the one hand, club theory helps to disentangle the complexity of different goods and processes in RCs and to diagnose resulting problems. Commons theory, on the other hand, is useful to retrace the principle and process through which collaboration problems can be solved.

### A Club Theory Perspective on RCs

Following club theory (Buchanan, 1965; Cornes and Sandler, 1996), a RC may be conceived of as a club, that is, as an interactive context that enables a group of actors, defined by their collaboration membership, to exclusively produce and consume a specific club good. With this theory, Buchanan emphasized the fact that there are further types of goods besides private and public goods (Sandler and Tschirhart, 1997). Club goods represent, in addition to private, public, and common goods, a fourth type of goods that is typically differentiated in economic theory (Ostrom et al., 1994). In contrast to private and common goods, they are not shaped by rivalry. This means the use by some does not make these goods less available to others. However, like private goods they are characterized by exclusiveness: Only club members are allowed to use its good. An everyday example is a golf club, with the exclusive club good of using a golf course (Buchanan, 1965). In the case of RCs, the specific club good is the joint production and use of an exclusive research opportunity (Baurmann and Vowe, 2014), which depends upon collaboration. Prototypically, this opportunity manifests itself in a joint application for third-party funding, in the research plan of a multi-disciplinary project group, or in the collaborative usage of research facilities. Of course, there may be other goods resulting from a RC that are non-exclusive, for example, knowledge. However, in our view, the exclusive research opportunity is the core good of a RC.

The club members' collaboration to produce and consume the good results in a series of specific problems, which can be traced back to the nature of a club good: Because it is collaboratively used by the club members, it should be equally available to all of them and at the same time be produced in a cooperative way (Baurmann and Vowe, 2014). Whenever this is not the case, one or several problems may occur. For example, the recurrent collaboration problem of fairness (e.g., Berlemann and Haucap, 2015; Bozeman et al., 2016; Johann et al., 2020) can be well-explained in terms of club theory: In the production and use of the club good, an appropriate reciprocity of input and output by and for everyone must be guaranteed. However, in a research team, there are also incentives for participants to limit their input at the expense of others or to take advantage of others. One important driving factor in this context is the principle of competition for authorship and reputation, which is deeply rooted in science. Therefore, self-interested behavior of individual participants may ultimately harm common interests. Another problem that can be traced back to the nature of a club is the issue of commitment (Baurmann and Vowe, 2014): A club always depends on the (long-term) investment of its members in order to work on a stable basis. This is not different for a RC, which depends on the input and efforts of all researchers involved.

### A Commons Theory Perspective on RCs

Commons theory (Ostrom, 1990, 2005) allows us to understand the principle and process through which collaboration problems can be solved. The crucial insight of this theory may be subsumed under the term self-governance: To best ensure a sustainable use and provision of their goods, communities are assumed to rely on self-regulation and not on other mechanisms like the state or the market (Ostrom, 1990). This includes not only defining suitable rules for the solution of potential collaboration problems but also successfully implementing and enforcing them. According to several case studies, this approach has proven to be superior to other governance mechanisms (Ostrom, 1990, 1999). Self-governance allows for an adjustment of the rules to local circumstances and increases the commitment of all community members and the binding character of their agreements.

Hence, through the lens of commons theory, RC is seen as an autonomous community that organizes and self-regulates the sustainable production and use of its good. It represents a useful framework for the study of RC because the principle of self-governance is consistent with academic practices and values, for example the self-administration of universities and the belief in freedom of science. Thus, the theory may be used to derive specific approaches for handling the good of a RC.

To summarize, in accordance with club theory, we define RCs as clubs which both produce and consume a specific club good (i.e., an exclusive research opportunity), which helps us to conceptualize potential collaboration problems. Commons theory allows us to understand the mechanisms of self-governance in collaboration and therefore to identify solutions for the occurring problems. The key to both understanding and addressing problems in collaborative research are, in our view, the experiences and expectations of RC participants. This concurs with an understanding of collaboration problems that has been outlined by Sacco (2020), who argued that failure of RC may result from unmet expectations of the researchers involved and thus from their individual experiences during collaboration. Therefore, these expectations can explain why collaboration might become problematic. Reconstructing the expectations of researchers involved in collaboration will facilitate our understanding of both problems and solutions.

### Summary of the Known (or Assumed) Problems and Solutions

The problems that we derived from club theory and from the various empirical findings are shown in Table 1.

**Table 1:** Problems in RCs as derived from theory and empirical findings.

**Problem**	**Source**
Fairness	Club theory (Buchanan, 1965)
Commitment	Club theory (Buchanan, 1965) and empirical evidence (e.g., Bozeman et al., 2016)
Difference	Empirical evidence (e.g., Bozeman et al., 2016)
Communication	Empirical evidence (e.g., Wöhlert, 2020)
Management	Empirical evidence (e.g., Volk, 2021)

An overview of the solutions is provided in Table 2 (selection of the most frequently mentioned findings).

**Table 2:** Solutions for problems in RCs as derived from theory and empirical findings.

**Solution**	**Source**
Basic principle of self-governance	Commons theory (Ostrom, 1990)
Competent leadership and management	Empirical evidence (e.g., Volk, 2021)
Effective and continuous communication, negotiation, and networking	Empirical evidence (e.g., Luo and Omollo, 2013)
Fostering commitment, trust, and a good relationship between collaborators	Empirical evidence (e.g., Shrum et al., 2007)
Provision of funding, incentives, and rewards as well as motivating participants	Empirical evidence (e.g., Wagner et al., 2019)
Working with previous collaboration partners	Empirical evidence (e.g., Hewitt-Dundas et al., 2019)
Optimal team size and composition	Empirical evidence (e.g., Mirnezami et al., 2020)
Working with partners with similar styles of working and objectives	Empirical evidence (e.g., Hara et al., 2003)

## Data and Methods

Our theoretical background helped us to take new perspectives as a starting point for our empirical investigation. As this innovative view calls for an exploratory procedure, we conducted a qualitative interview study. In our study, we followed a deductive-inductive approach with the central objective of testing and iteratively developing a set of analytical categories for problems and solutions in RC. The deductive categories were based on the problems and solutions derived from theory and empirical evidence (see Tables 1, 2). The inductive categories were then developed on the basis of the interviews we conducted, aiming (1) to differentiate and add depth to the known/assumed problems and (2) to identify new problems and solutions. This process continued until the researchers observed that problems and solutions reached the state of theoretical saturation, meaning that as the analysis proceeded, the material yielded no substantial further differentiations or new problems/solutions.

### Study Population

The study included 18 in-depth interviews with 19 academic researchers from seven collaborative research teams in Germany. We selected the teams so as to ensure the coverage of a wide range of disciplines (including interdisciplinary teams) as well as different team sizes and constellations (including both academic-only and university-industry collaboration teams). The key criteria for sampling were the degree of (1) disciplinary and (2) organizational heterogeneity. Based on the number of science areas represented in the RC (Humanities, Social Sciences, Engineering, Natural Sciences, Life Sciences) and the number of organization types (universities, universities of applied sciences, research institutes, large enterprises, SME, NGO, …), we calculated a heterogeneity score. This score ranged from 2 (a small RC involving only university researchers in the same field of science) to 7 (RC including 3 different fields of science and four organization types). Table 3 shows the seven different RCs, the disciplines involved, and their heterogeneity score.

**Table 3:** Key characteristics of the seven RCs under investigation.

**Research theme**	**Fields of research**	**Organization types**	**Heterogeneity index**
Heart valve infections	Life Sciences (1)	Universities (1)	2
Crop science	Life Sciences (1)	Universities, research institutes (2)	3
Robots in logistics	Engineering (1)	University, SME, large enterprise (3)	4
Sustainable traveling	Social Sciences (1)	University, SME, industry association (3)	4
IT security	Engineering, Humanities, Social Sciences (3)	University, university of applied sciences (2)	5
Methane as fuel	Engineering (1)	Universities, universities of applied sciences, research institutes, large enterprises, SMEs (5)	6
Water treatment	Natural Sciences, Engineering, Social Sciences (3)	Universities, federal authorities, large enterprises, SMEs (4)	7

*Based on the number of science areas represented in the RC (Humanities, Social Sciences, Engineering, Natural Sciences, Life Sciences) and the number of organization types (universities, universities of applied sciences, research institutes, large enterprises, SME, NGO, …), we calculated a heterogeneity score with a minimum of two points (one science area, one organization type)*.

From each team, we interviewed two or three researchers with different roles: speakers (*n* = 7), principal investigators (*n* = 8), and in some cases managing coordinators (*n* = 4). In one case, a speaker and a coordinator were interviewed together; therefore, the total number of informants (*N* = 19) is higher than the number of interviews.

### Interview Protocol

Two authors of this paper conducted the interviews, some jointly and others individually, between April 2019 and May 2020. While in 2019 the interviews were conducted face-to-face (*n* = 9), the COVID-19 pandemic forced us to switch to online interviews in 2020 (*n* = 9). The interviews were based on a method developed by Gläser and Laudel (2009); see also Laudel and Gläser (2008) and lasted between 70 and 135 min. In total, the recorded interviews had a duration of 27:39 h. After some opening questions, we asked the respondents to report on the problems we suggested as well as further problems, and on approaches to solve these problems. These questions were derived based on the problems and solutions which we identified through the theoretical foundation of our study and the above-mentioned state of research. The protocol was developed iteratively, integrating new problems and solutions found in previous interviews within our study. In order to achieve a common understanding of the problems and possible solutions, problems were described in detail and examples discussed in the interviews. Evidence of the mutual understanding is that the interviewees each provided their own examples of the problems, some of them new, which allowed us to include new problem dimensions. At the end of the interview, the interviewees were invited to mention any aspects that they additionally deemed important for collaborative research.

### Extraction and Organization of Interview Data

Problems and solutions that were derived from previous research were also used to develop a set of categories for the extraction and organization of the interview data. The interviewer who participated in most of the interviews also conducted the entire coding based on the transcribed interviews. The problem and solution categories were iteratively elaborated and refined throughout the coding process and the detailed analysis (see Laudel and Gläser, 2008; Gläser and Laudel, 2009). In this way, we were able to increase the granularity of our categories and, for instance, add different variants of problems as sub-categories. Additionally, we found a range of new problems and solutions, which we included in our set of categories and in the subsequent analyses. Based on the problems discussed by the interviewees, we reconstructed their expectations of how a research collaboration should look, including suitable methods to solve the respective problem.

In a further step, based on the frequency of mentions by the interviewees and the ascribed importance, we weighted the problems. For this purpose, we categorized the importance of each problem mention as high/medium/low based on the explicit or implicit weighting by the interviewee. A mention of a problem ascribed with high importance was assigned five points, a problem with medium importance was given three points and low importance one point. To further emphasize a problem's ascribed relevance, we added another point for each mentioned solution that could be attributed to the same problem. Based on the resulting score, we classified the problems as highly important, somewhat important, and less important.

## Results

Below, we present our findings in two main parts: First, we outline the problems which researchers perceive with regard to RC (RQ1). Subsequently, we present a set of solutions which researchers perceive as useful to solve problems in collaborative research (RQ2).

### Seven Main Problems in RC

As explained in the theoretical section, a RC may be seen as a club, with the specific club good in the present case being the production and use of an exclusive research opportunity. Based on this approach, we concluded that problems occur if members believe that they cannot benefit from their club membership as expected. Put differently, collaboration becomes problematic if the current practice substantially differs from participants' expectations toward RC, which can be traced back to one overarching expectation, that is, of a successful and productive collaboration. We identified seven problems, while each one affects a specific expectation toward collaboration: difference, commitment, certainty, communication, fairness, management, and personal relationships. We explain these problems and their specific differentiations, which we developed based on the empirical material, in the following. The ascribed relevance of the problems differs between various types of collaboration; the order depicted in Table 4 reflects the aggregate view of all our interviewees taken together. It is to be understood as a tentative problem hierarchy that needs further testing and validation, preferably by means of quantitative measures. New problems that have not been described yet in the literature or which could not be derived from theory were highlighted in bold.

**Table 4:** Seven main problems of research collaboration.

High relevance	Difference problem Commitment problem **Certainty problem**
Medium relevance	Communication problem Fairness problem Management problem
Low relevance	**Relationship problem**

*Depending on the prominence and the detail the interviewees ascribed to the different problems, we assessed whether they were ranked high (three points for each mention), medium (2) or low (1 point). Additionally, we gave one point for each solution that was mentioned to tackle one of the seven problems. The resulting score allowed us to tentatively weight the problems. Highlighted problems are new problems that were developed from the empirical analysis*.

#### Difference Problem

The most important problem occurs if the difference between members of a RC is perceived as too large. This problem is based on the expectation that differences within a RC should be smaller or bridged effectively. One example is different perspectives of collaborative partners, as mentioned by an engineer from a large-scale enterprise:

“Well, universities sometimes act like know-it-alls. [...] In some cases, they are not really compatible with my world. [...] Universities often have their own perspective, which is very theoretical, and one has to bring this into line and they need to be intrigued.”

Our interviews revealed the following differentiation into three aspects of difference:

- differences with respect to motivations and objectives, specifically concerning the area of conflict between strategic and substantial interests
- cognitive differences, for example concerning scientific foci and styles of working
- social differences like gender or organizational diversity

The difference problem is notorious for, but not exclusive to, interdisciplinary and university-industry collaborations. A possible consequence is conflicts among collaboration partners, for example between academic members who want to publish research findings as soon as possible and corporate members who first want to claim a patent. If difference is not bridged effectively, the productivity of the collaboration may be hampered.

#### Commitment Problem

Collaboration in a RC can be perceived as problematic if participants consider the commitment of a substantial proportion of the research team to be too low, which is the case when they notice that others are rather focusing on their own research domain at the cost of the collective interests of the RC. This problem relates to the expectation that a higher level of commitment of all participants is necessary. Such commitment affects the RC as a whole and thus refers to the stress ratio between particular and collective interests. An example of this problem would be an exploitation of a RC in financial terms, as emphasized by an engineering researcher:

“And so, it was quite clear that some groups tried to hide behind the consortium. That means they pulled out some money for their research. I think they did many other things with this money.”

The commitment problem can be differentiated according to the specific interests of a RC. Collaboration can thus become problematic if participants perceive that their collaborators:

- do not sufficiently ensure the intellectual coherence of the individual parts of a RC (i.e., work groups, subprojects) and cooperate beyond internal dividing lines
- do not get involved in cross-sectional tasks of a RC, e.g., a graduate school
- are committed on a short-term rather than on a long-term basis

The commitment problem is first and foremost a threat to the overarching expectation that collaborative research should be productive and meet its goals. Furthermore, it creates uncertainty because if involved partners do not deliver their contribution (in time), the whole collaborative process might be severely affected.

#### Certainty Problem

Although previous research suggests that uncertainty is often perceived as a scientific routine and a prerequisite for successful research (e.g., Whitley, 1984; Shrum et al., 2007), we found that a problem arises if participants perceive a lack of certainty to be a burden to the collaboration. According to our interviewees, avoidable uncertainties can lead to unforeseeable burdens. For instance, a representative of a small-enterprise tourism researcher described how it affected the collaboration when a leading partner dropped out: “If decision-makers change during the project, [...] that is a medium-level disaster. Because handovers do not take place. The information from the predecessor, what he discussed with the project participants, is not available.”

The underlying expectation which can be reconstructed from this problem is that uncertainties and risks in collaboration should be minimized as far as possible. Contrary to the problems mentioned above, we also found empirical evidence for the opposite situation: Rigid and strict rules and requirements can impose constraints on collaborative researchers, which can also be perceived as problematic.

The certainty problem can be differentiated based on the source of (un-)certainty:

- funding institutions, e.g., denial of a funding extension
- collaboration partners, e.g., delayed responses or premature exit
- employees or individual members, e.g., premature termination of contracts

The certainty problem is always a latent threat to the success of a RC. While some unforeseen developments may be compensated by agile leadership, other events such as the dropout of a collaboration partner can have a negative effect on the success and productivity of the collaboration. The latter case typically puts both leadership and members under substantial stress because processes are delayed or not all research goals can be met.

#### Communication Problem

Another problem occurs if participants of a RC perceive insufficient information and discussion between leadership and membership such that members cannot adequately participate in decisions. The underlying expectation denotes that communication processes should be of a reciprocal nature. A biologist described a situation in which members felt a lack of information from their leadership: “I think that's a reason when things don't work out. If people do not understand how decisions come about; when they feel left out because they don't have all the information.”

We can differentiate this problem based on who is perceived to be responsible:

- leadership does not communicate appropriately, provides too little information and space to articulate the needs of members, does not listen or include members in discussions
- membership does not communicate appropriately, does not listen, engage in discussions or inquire about information

When members of a RC consider internal communication of either leadership or membership to be inappropriate, they refer to different aspects. Most importantly, the informational value and the choice of communication channels are mentioned. When a communication problem arises, it often leads to discontent and the impression that decision-making is intransparent. This can also negatively affect trust in leadership or in other members.

#### Fairness Problem

Collaboration can also become problematic if participants perceive the distribution of individual inputs and outcomes to be unfair. Fairness is conceived of as a state in which the input of each participant is in reasonable proportion to the individual outcome, and that the ratio of input and outcome is similar among all participants of a RC. According to a leading biologist at a university, fairness problems “often arise from conflict concerning authorships. Who actually has the greater intellectual stake in a joint project?”

Empirically, we found three differentiations of this problem according to three central resources that constitute the returns of a RC:

- decisions about lead authorship in publications
- distribution of personal, financial and technical resources
- receipt of recognition and appreciation

If members perceive a lack of fairness, it typically has a detrimental effect on the personal relationships within the collaboration, and further leads to frustration among those individuals who consider themselves to be at a disadvantage.

#### Management Problem

The management problem occurs if members of a RC notice incompetence in their leadership. This relates to the expectation that the leadership of a RC should be qualified beyond academic expertise and able to manage the collaboration successfully and efficiently. As an IT security researcher noted self-critically about a structured program for doctoral students managed by himself and other researchers, “we overregulated [...] that a bit, I thought. [...] they had to do too much. Yet another symposium, another workshop and another seminar and this and that.”

There are three differentiations of this problem, which refer to the relationship of an RC's management to the other actors involved. Accordingly, collaboration can become problematic if participants feel that researchers in leading positions:

- take advantage of their status rather than focusing on the interests of the RC as a whole
- tend to restrict the autonomy of individual members too much or apply a management style without guidance
- are not capable of dealing flexibly with the requirements laid out by the funding institutions

According to our interviewees, the management problem can aggravate other problems. For instance, when the management of a RC is not sufficiently competent to take steps to reduce a sense of unfairness in the RC or to bridge cognitive differences effectively, it can be detrimental to the success of collaboration and also lead to frustration among members.

#### Relationship Problem

Collaboration can also be perceived as problematic if personal relationships among participants tend to strain and hinder work processes rather than supporting and stimulating research. As a medical researcher put it, “The biggest problem is when you can't stand people in a research network. [...] Because would you sit together with someone [...] if you didn't like the person at all? You won't sit down in the evening with him in a pub and brainstorm over dinner.”

From this problem, we can reconstruct participants' expectation that personal relationships should be more beneficial or at least less detrimental to collaborative research. We can differentiate this problem into two contrasting variants. Relationship problems occur if participants perceive:

- personal relationships as too tight and demanding
- social distances as too great, which does not allow for personal relationships based on mutual liking

Some of our interviewees stated that in the case of problematic social relationships, collaboration might be less productive, while others did not attribute particular relevance to this problem.

### Solutions to Problems in RC: Three Perspectives on Self-Governance

To identify solutions to problems that researchers perceive as useful, we sought to carve out what can be done to meet the overarching expectation of a productive and successful collaboration. To draw conclusions about solutions that are generalizable beyond specific contexts, the participants' expectations and experiences are paramount in as much as they act as hinges between problems and solutions: Collaboration becomes problematic if the current practice deviates substantially from expectations. Thus, the function of solutions is to converge the current practice toward the expectations of participants.

At this point, we need to emphasize that the basic principle of solutions is neither a laissez-faire policy nor governance from the outside or from superior institutions (e.g., university or politics). Rather, based on the insights of commons theory, we suggested self-governance as the basic principle to address problems in collaborative research. The most important question refers to the rules themselves, that is, what can be done to solve the problems outlined above. Two further questions are also of relevance, albeit less so: Who is responsible for solving collaboration problems? And when should collaboration problems be addressed?

#### What? 12 Preferred Solutions for Self-Governance

From our empirical insights, we were able to extract 12 solutions, which are regarded as more or less suitable to address collaboration problems and to converge the current practice toward the participants' expectations. We analyzed the interviewees' answers and grouped their suggestions according to the central targets which we reconstructed from the mentioned solutions: (1) the participants of a RC and their composition, (2) the cognitive basis of a RC, i.e., participants' shared knowledge, standards, and values, and (3) interaction and communication within a RC. The resulting set is summarized in Table 5. New solutions that have not been described as such in the previous literature or could not be derived from theory are highlighted in bold letters.

**Table 5:** Twelve solutions to collaboration problems.

**Solution**	**Description**	**Exemplary problems to address**
**Target: Participants**		
1. Selection of Participants (proactive)	Selection based on proven ability to collaborate, e.g., previous collaboration partners, as a means to reduce risks for the collaboration and increase time efficiency	Commitment problem, certainty problem
2. Motivation (reactive)	Motivation and appreciation of all participants, from principal investigators to doctoral students, e.g., by providing incentives or appealing to the individual interests of the participants	Commitment problem, fairness problem
3. Leadership personality (proactive)	Integrative and competent personality for leadership combining experience, authority and pronounced communicative capabilities	Management problem, relationship problem
4. Personal relationships (proactive)	Trust-building by maintaining personal relationships, e.g., informal meetings	Relationship problem, communication problem
**Target: Cognitive basis**
5. **Research program** (proactive)	Development of a research program that integrates the interests and competencies of all participants, including a joint definition of research goals, to secure a high commitment by all participants	Difference problem, commitment problem
6. **Common ground** (proactive)	Creating common ground, e.g., through collaborative verbalization of a self-concept (common identity), methodological norms, or a compelling research idea inspiring the joint research, e.g., in interdisciplinary contexts	Commitment problem, difference problem
7. **Set of rules** (proactive)	Joint development of a codified set of rules for the collaboration incl. dos and don'ts, as a means to both reduce and resolve conflicts, e.g., with regard to disclosure of results	Fairness problem, certainty problem
**Target: Interaction and communication**		
8. **Appropriate style of leadership** (proactive)	Leadership style that is adjusted to the type of research collaboration, ranging from participatory (high autonomy of members) to centralized leadership (low autonomy), depending on both the size of the RC and the organizational cultures involved	Management problem, difference problem
9. **Communication space** (proactive)	Creating and using a shared communication space, e.g., online collaboration tools but also offline venues for in-person exchange, to increase transparency and create opportunities for low-threshold participation	Communication problem, fairness problem
10. **Handling of conflicts** (reactive)	Constructive handling of conflicts by explicating and integrating e.g., different research interests, disciplinary perspectives or methodological standards	Difference problem, relationship problem
11. Synchronization (proactive)	Synchronization of processes through the determination of deadlines, tasks, and responsibilities	Certainty problem, management problem
12. **Evaluation** (reactive)	Continuous evaluation of collaboration, including the detection of conflicts or problems through listening to the needs and concerns of members and control of target achievement	Management problem, commitment problem

*Highlighted solutions are new solutions developed from the empirical analysis*.

This eclectic set of possible solutions to collaboration problems represents the rules for self-governance of a RC. However, two central points need to be emphasized: First, our interviewees' experience shows that none of the problems can be solved through one single solution, and none of the solutions accounts for one specific problem. Rather, every single problem requires a balanced set of different solutions that is based on extensive negotiations between all affected participants in order to reflect their individual viewpoints, needs, and preferences. For instance, the difference problem can be addressed on a cognitive basis by a research program that integrates the various disciplinary perspectives represented in a collaboration, for instance by integrating the interests and competencies of all participants, including a joint definition of research goals (Solution 5). The same problem, however, can also be addressed on an interactional level by creating opportunities to strengthen social ties within or outside the typical venues of the collaboration (Solution 4). Both solutions can of course also be applied in a complementary manner—a principle that is often stressed by our interviewees, as one solution is often not considered sufficient for tackling a problem effectively. Nevertheless, we also found some general tendencies during the course of our interview analyses: For example, as solutions to the commitment problem, Solutions 1 and 6 (selection of participants and common ground) were predominantly mentioned, while for the management problem, Solutions 8 and 11 (appropriate style of leadership and synchronization) were often emphasized.

Another central point relates to the fact that the listed solutions are not equally applicable to all kinds of RCs. This specifically becomes apparent through Solution 8, for which we recommend, based on our interviews, different variations depending on the type of collaboration. But it also affects the selection of solutions in general. While for one RC an occurring commitment problem might be solvable by establishing a common ground (Solution 6), for another RC the strengthening of personal relationships might be more efficient (Solution 4).

Taken together, these two points underline that the varying conditions need to be considered. On the macro level, this mainly affects the societal environment and science policy regulations. Financial resources and university politics are examples of relevant conditions on the meso level. Lastly, on the micro level, different characteristics of the researchers involved in collaboration have been mentioned, for example different styles of working or motivations. Moreover, the phase of the collaboration needs to be taken into account when selecting solutions. Problems that occur in advance (i.e., while working on the application) call for different solutions than problems that occur during or after the collaboration. For example, the commitment problem might be solved by selecting reliable participants in advance (Solution 1), while it might be advisable to solve this problem by creating a common ground (Solution 6) once the collaboration has started.

#### Who? Actors of Self-Governance

With respect to the question of who is responsible for solving collaboration problems, we cannot extrapolate general recommendations from our interviews. Rather, different constellations along a spectrum are possible, ranging from a pronounced focus on leadership to the involvement of all participants. The former type is recommended for RCs with centralized leadership, often to be found in university-industry collaborations. Meanwhile, university-only RCs tend toward more participatory forms of leadership. A variant of this approach, suitable for larger collaborations, is an elected steering committee representing all members. Due to the heterogeneity of participants in a RC, there are always different perspectives involved, which leads to different ideas of who should be responsible. Therefore, like the solutions themselves, this needs to be carefully negotiated within the self-regulatory framework of RCs. The level of consensus between the participants then results in the decision about how urgently which actors need to take action.

#### When? Strategies of Self-Governance

Finally, we aim to answer the question of when collaboration problems should be solved, which relates to self-governance as a process. Primarily, this concerns different strategies and we can basically differentiate between a reactive and a proactive strategy: Problems can be addressed once they occur, for example motivating participants (Solution 2) might help to solve a commitment problem that develops over time. Alternatively, problems can be foreseen and possibly prevented, for example the relationship problem might be averted by selecting suitable participants for a research team (Solution 1) from the very outset. From our interview data, we cannot derive a specific pattern. Instead, there are hybrid forms of both strategies with a slight dominance of the proactive one.

## Discussion

### Summary of Our Findings

The aim of this study was to answer the following questions:

RQ1: Which problems do researchers perceive with regard to RC?

RQ2: Which problem solutions do researchers within RC perceive as useful?

To answer these questions, we relied on a theoretical framework that combined two micro-oriented approaches: club theory and commons theory. Based on the notion that problems are perceived where participants' expectations toward the collaboration are not met, we found that problems may occur in RCs with respect to seven aspects: commitment, difference, commitment, certainty, communication, fairness, management, and personal relationships. The three most important problems are the difference problem, which results from too large differences with respect to the composition of a RC, the commitment problem, which occurs if participants focus on their individual interests at the expense of collective interests, and the certainty problem, which is caused by unpredictable uncertainties and risks in collaboration. In many cases, these problems co-occur and mutually cause each other. The management problem, for example, can exacerbate an existing commitment and/or a certainty problem. Furthermore, the problems and their particular manifestation depend on the specific context, for example the phase of collaboration or the type of partners involved.

Based on our empirical insights, we developed a set of 12 solutions which have three targets: the participants of a RC, the cognitive basis of a RC, and the interaction and communication within a RC. These solutions may, from the interviewees' perspective, help to address or prevent collaboration problems. Their central function lies in the convergence of the current practice toward the expectations of participants. Based on this understanding of problems and solutions, it is possible to realize self-governance as an effective way of organizing RCs, defined as research units working on a joint project.

### Theoretical and Practical Contributions

Several of the problems and solutions we identified resonate with previous scholarship on collaborative research. With respect to collaboration problems, for example, various scholars have emphasized that differences among participants (e.g., Barnes et al., 2002; Bozeman et al., 2016; Volk, 2021) or a lack of commitment of participants (e.g., Barnes et al., 2002; Gaskill et al., 2003; Bozeman et al., 2016) can be obstacles to joint research, which resembles what we called the difference and the commitment problem. Previous research has also mentioned some of the solutions we suggested, such as fostering trust through good personal relationships (e.g., Chompalov and Shrum, 1999; Shrum et al., 2001, 2007; Bruneel et al., 2010) or the assignment of a competent leadership (e.g., Vonortas and Spivack, 2006; Schützenmeister, 2010; Volk, 2021). However, to the best of our knowledge, no previous study has provided a similarly systematic picture. Furthermore, our study differentiated and expanded our knowledge of collaboration problems and ways in which to solve them. We were able to advance an in-depth understanding of problems in RCs and identify different dimensions of each problem. Moreover, we also identified uncertainty as a problem which, probably because it has been suggested as a common standard of research (e.g., Whitley, 1984; Shrum et al., 2007), has received barely any attention in the context of collaborative research so far. Furthermore, we strengthened the importance of problems that have been mentioned before and specified their implications, for example with respect to the fairness problem. With regard to a self-governance perspective, we found several new solutions (see Table 5), such as the development of an integrative (Solution 5) creating cognitive common ground between participants (Solution 6), and the continuous evaluation of the collaboration (Solution 12).

Considering these achievements, we conclude that the micro-theoretical lens and the unique combination of club theory and commons theory is a useful and productive framework for the analysis of RC. In particular, club theory helped us to identify the problems that result from a target-oriented association of researchers and to strengthen problems which had not received much attention before, for example, the fairness problem. Commons theory allowed us to establish a useful framework of academic self-governance that is sustainable and sensitive to the specific conditions of collaborative research. Thanks to their combination, these approaches helped us to reduce the tension between individual and collective interests. Furthermore, both theoretical approaches, and more importantly, their combination sets a clear focus on the interaction of researchers as both basis for problems and solutions. This understanding of governing collaborative research is consistent with current developments in science and technology studies: Using the term “tentative governance,” scholars have most recently suggested to rely on a form of governance that is dynamic, flexible, and sensitive to the respective conditions and requirements of the scientific environment rather than on definite and persistent forms (Kuhlmann et al., 2019). Additionally, our study lends support to the assumption that individual expectations influence not only the perception but also the relevance of collaboration problems and the preference for specific solutions. Including this subjective aspect might, in our view, be a relevant advancement for both theories upon which we relied.

Furthermore, previous research mainly focused on one type of RC (e.g., university-industry collaboration) or specific problems and solutions. As we believe that the presented catalogs are generalizable beyond single contexts while at the same time being concise, they promote a consistent systematization of this field and may be used as heuristics for further empirical studies on collaborative research (e.g., surveys). In addition, they enhance our understanding about the success or failure of collaborative research and may serve as a foundation for practical conclusions, for example as guidelines for consulting and coaching. Lastly, the problems and solutions we found for RCs may also inform similar studies on other forms of scientific collaboration such as co-authorship or cooperation within scientific organizations.

### Limitations and Gaps for Future Research

A first limitation refers to the fact that we did not consider objective parameters for the success and performance of RCs, such as bibliometric measures. Rather, the problems we identified were solely based on the participants' experiences and perceptions, and the solutions they suggested cannot be evaluated with respect to their objective effectiveness. Thus, further validation is needed. Second, because our sample included (purportedly) functioning RCs only, there might be more important or more serious problems in collaborative research that our catalog is missing. Third, because we took a general look at problems and solutions we did not differentiate between different types of RCs, for example in terms of their size (i.e., number of researchers and organizations) and constellation (university-industry vs. academic-only), the type of funding, or the phase of collaboration. The latter one specifically has important consequences for collaboration problems as well as solutions (Volk, 2021). And it may well be the case that some problems only occur in specific forms of RCs and/or vary in their intensity depending on the type of RC. Fourth, while we emphasized that the varying conditions of collaboration are relevant, we did not investigate these conditions in depth. Therefore, further empirical studies are needed in order to identify the relationships between the conditions of collaborative research, potential problems, and useful solutions. Fifth, we focused on German RCs in our study. Although we assume that the identified problems also occur in RCs in other countries and cultures and that the solutions are similarly applicable, further research is needed to ensure the generalizability of our catalogs beyond this and a solely national context. Sixth, we focused on the viewpoints of speakers, principal investigators, and coordinators. Our sample did not include further members of RCs such as postdocs and doctoral students, which might have led to additional insights.

## Conclusion

Our study offers a theoretically and empirically informed, systematic framework for understanding and addressing problems in collaborative research. This framework may help to maximize the prospects of success for all sorts of scientific collaboration. However, our study is only a starting point. We thus hope to inspire further research that refines and extends our insights. Most specifically, future studies, for instance quantitative surveys, can apply and validate our catalogs of problems and solutions with respect to collaboration in different disciplines and countries, and to different sizes and composition of RCs, in order to verify whether researchers are able to prevent problems and ultimately the failure of collaboration as far as possible.

## Data Availability Statement

In April 2022 our data will be published under the following doi: https://doi.org/10.21249/DZHW:decquali:1.0.0.

## Ethics Statement

Ethical review and approval was not required for the study on human participants in accordance with the local legislation and institutional requirements. The patients/participants provided their written informed consent to participate in this study.

## Author Contributions

FM and GV contributed to conception and design of the study. FM organized the database and performed the analysis. FM and CW wrote the first draft of the manuscript. All authors contributed to manuscript revision, read, and approved the submitted version.

## Funding

This research was supported by the German Federal Ministry of Education and Research (Grant Number: M527800).

## Conflict of Interest

The authors declare that the research was conducted in the absence of any commercial or financial relationships that could be construed as a potential conflict of interest.

## Publisher's Note

All claims expressed in this article are solely those of the authors and do not necessarily represent those of their affiliated organizations, or those of the publisher, the editors and the reviewers. Any product that may be evaluated in this article, or claim that may be made by its manufacturer, is not guaranteed or endorsed by the publisher.
